# Polymorphisms in *SHISA3* and *RFC3* genes and their association with feed conversion ratio in Hu sheep

**DOI:** 10.3389/fvets.2022.1010045

**Published:** 2023-01-05

**Authors:** Changchun Lin, Weimin Wang, Deyin Zhang, Kai Huang, Xiaolong Li, Yukun Zhang, Yuan Zhao, Jianghui Wang, Bubo Zhou, Jiangbo Cheng, Dan Xu, Wenxin Li, Liming Zhao, Zongwu Ma, Xiaobin Yang, Yongliang Huang, Panpan Cui, Jia Liu, Xiwen Zeng, Rui Zhai, Landi Sun, Xiuxiu Weng, Weiwei Wu, Xiaoxue Zhang, Wenxin Zheng

**Affiliations:** ^1^College of Animal Science and Technology, Gansu Agricultural University, Lanzhou, China; ^2^The State Key Laboratory of Grassland Agro-Ecosystems, College of Pastoral Agriculture Science and Technology, Lanzhou University, Lanzhou, China; ^3^Institute of Animal Science, Xinjiang Academy of Animal Sciences, Ürümqi, Xinjiang, China; ^4^Institute of Animal Husbandry Quality Standards, Xinjiang Academy of Animal Sciences, Ürümqi, Xinjiang, China

**Keywords:** association analysis, *SHISA3*, *RFC3*, feed conversion ratio, tissue expression, Hu sheep

## Abstract

In animal husbandry, feed efficiency is a crucial economic trait. In this study, the general linear model was used to perform association analysis for various genotypes and feed conversion ratio (FCR)-related traits. Reverse transcription-quantitative PCR (RT-qPCR) was used to detect the expression of *SHISA3* and *RFC3* mRNA levels in 10 tissues from 6 sheep. The results showed that SNPs in the NC_040257.1:c.625 T > C and NC_040261.1:g.9905 T > C were analyzed whether they were associated to feed efficiency parameters in Hu sheep (body weight, feed intake, average daily growth, and feed conversion ratio). NC_040257.1:c.625 T > C was shown to be significantly associated with body weight at 80, 100, and 120 days as well as feed conversion ratio (*P* < 0.05), whereas NC_040261.1:g.9905 T > C was found to be significantly associated with average daily weight gain from 80-140 days (ADG80-140) and FCR (*P* < 0.05). In Hu sheep, the CC genotypes of SHISA3 and RFC3 were the most common genotypes related to feed efficiency traits. Furthermore, the feed conversion ratio of the combined genotypes TT^*SHISA*3^-CC^*RFC*3^, TT^*SHISA*3^-CT^*RFC*3^, TT^*SHISA*3^-TT^*RFC*3^, CT^*SHISA*3^-CC^*RFC*3^ and CT^*SHISA*3^-CT^*RFC*3^ was significantly better than the FCR of CC^*SHISA*3^-TT^*RFC*3^. RT-qPCR results showed that the expression levels of *SHISA3* were lower in the lung than in spleen, kidney, muscle and lymph (*P* < 0.05), and RFC3 was the lung had a highly significant higher expression level than the heart, liver, spleen, and muscle (*P* < 0.01). In conclusion, *SHISA3* and *RFC3* polymorphisms can be used as genetic markers for improving feed conversion efficiency in Hu sheep.

## 1. Introduction

Sheep farming plays a vital role in the regional economic development of Northwest China. According to studies, the cost of feed accounts for between two-thirds to three-quarters of the entire cost in a housed sheep farming system (1). Improving the efficiency of sheep feed consumption not only increases farmer income, but also decreases methane gas emissions for environmental protection (2). The feed conversion ratio (FCR) is a traditional metric for assessing feed efficiency (FE), which is defined as the ratio of feed intake to body weight (BW) growth per unit time (3, 4). Therefore, improving FE has become a major concern for sheep farmers. With the fast advancement of science and technology, whole genome sequencing is now extensively employed in livestock and poultry breeding, including pigs (5–9), cattle (10–13) and other species (14). Many relevant candidate genes and quantitative trait loci associated to feed efficiency were screened using sequencing technology, which may be used for marker-assisted selection (MAS) to enhance FE and lower production costs (15–21). In Hu sheep, it was discovered that *ME1* gene polymorphism was significantly associated with FCR and RFI (*P* < 0.05), whereas *CA1* gene polymorphism was significantly associated with FCR (*P* < 0.05) and the polymorphism in gene *RTP4* were significantly associated with RFI (*P* < 0.05) (17, 22). According to methodological research using genome-wide association analysis, the genes *CREB1, STEAP4, CTBP1, RIP140, SMURF2, FBF1, DTNBP1, SETD7*, and *RBM11* may be candidates for fat deposition in sheep tails (23, 24). According to research, synonymous mutations in *ELOVL5* are associated to tail width, tail fat weight, and relative tail fat weight (*P* < 0.05). *FASN* synonymous mutations were shown to be related to tail length and breadth (*P* < 0.05) (25).

In this work, we selected two candidate genes, *SHISA3* and *RFC3*, which showed differential expression in previous studies on residual feed intake (21), while circulating RNAs associated with lamb feed efficiency were identified as miRNA target genes in studies (22). *SHISA* family member 3 (*SHISA3*) is a gene that codes for proteins. *SHISA3* belongs to the *SHISAs* family of endoplasmic reticulum-resident proteins, which has eight members (*SHISA2*–*SHISA9*) and is mostly involved in head development in non-human animals such as Xenopus, mouse, and chicken (26–28). *SHISA3* research is mostly focused on human cancer. *SHISA3* has been found to be a tumor suppressor gene, inhibiting carcinogenesis, invasion, and metastasis by increasing—catenin degradation (29). *SHISA3* gene expression has been shown to be dysregulated in colorectal cancer (30), laryngeal squamous cell carcinoma (31), and nasopharyngeal carcinoma (32) in subsequent research. This demonstrates the crucial role of *SHISA3* in clinical tumor identification and prediction. However, the effect of *SHISA3* gene with sheep FE is not clear. The eukaryotic replication factor C (*RFC*) complex is part of the DNA polymerase, which consists of five subunits (*RFC1-5*) (33, 34). It functions as a AAA^+^ ATPase that is required for DNA replication, damage repair, and cell cycle checkpoint regulation in all eukaryotes (35–40). The RFC is a clamp loader that helps to lengthen the DNA strand by loading proliferating cell nuclear antigen (PCNA) onto primed DNA (41). The replication factor C subunit 3 (*RFC3*) is a subunit of the RFC complex. Reduced *RFC3* expression has been shown to inhibit cancer cell proliferation by forming complexes with proliferating cell nuclear antigen (PCNA) (42). *RFC3* has recently been described to be largely focused on human malignancies, such as liver, breast, esophageal, and ovarian cancers, where it plays a significant role in cell proliferation, invasion, and metastasis (42–45). However, the effect of *RFC3* on animal FCR remains unclear.

Thus, the following hypotheses were proposed in this study: (1) the presence of polymorphisms in the *SHISA3* and *RFC3* genes; (2) the different genotypes of the polymorphic loci are associated with feed efficiency in sheep. To test the validity of the hypothesis, the purpose of this study was to identify *SHISA3* and *RFC3* polymorphisms, to associate different genotypes of polymorphic loci with feed efficiency attributes in sheep, and to examine *SHISA3* and *RFC3* expression levels in various tissues of sheep.

## 2. Material and methods

### 2.1. Ethical statement

All animal experiments were conducted out in compliance with the rules and recommendations of Gansu Province's NPC government and were authorized by Gansu Agricultural University's Animal Health and Ethics Committee (Animal Experimentation License No. 2012-2-159).

### 2.2. Animal management, data collection, and DNA isolation

In this research, 1,382 male Hu sheep lambs were obtained from Defu Agricultural Technology Co., Ltd (Gansu, China). Their birthplaces were from different farming enterprises, and the detailed sources refer to the study of Lin et al. (46). Before weaning at 56 days of age, all of the lambs were in excellent development condition and fit, and they had all finished a standardized vaccination schedule administered by a practicing veterinarian. After weaning, the lambs were transported in batches to Defu Agricultural Technology Co., Ltd. and housed inside in separate 0.8 × 1 m enclosures until the trial ended. All lambs were given the same feed and were subjected to the same management settings as in our prior research (1). It can be briefly summarized in three phases: a 14-day transition period, a 10-day acclimation period and a 60-day formal experiment. The transition phase consisted of transitioning from traditional feed to 100 percent pellet feed and then maintaining it until the conclusion of the trial, during which time the feed and water supply was enough to fulfill the lambs' needs for free feeding and drinking. The particles used in the experiment were all from Gansu Sanyang Jinyuan Animal Husbandry Co., Ltd (Gansu, China). The weight of lambs was recorded at 80 days of age as the starting weight, and then every 20 days until the lambs were 140 days of age as the ending weight, while feed consumption was collected for the calculation of average day feed intake (ADFI), average daily gain (ADG), and FCR.


$$\begin{array}{l} ADG=\;\frac{\left(BWf\;-BWi\right)}{N}\\ ADFI=\frac{\;total\;feed\;consumption}{N}\\ FCR=\frac{ADFI}{ADG} \end{array}$$

where BWf represents 140 days weight; BWi represents 80 days weight; N represents number of experimental days. After the experiment was completed, a blood sample (5 ml) was taken from each lamb's jugular vein for further DNA extraction. Genomic DNA was extracted from 1,382 blood samples using the EasyPure Blood Genomic DNA Kit (TransGen Biotech, Beijing, China), dissolved in TE buffer, and kept at −20°C according to the manufacturer's recommendations.

### 2.3. SNPs identification and genotyping

Using the Oligo 7.0 software (Olgi.net, Colorado Springs, CO, USA), specific PCR primers (GenBank Accession Nos. NC_040257.1 and NC_040261.1) were based primarily on the genomic DNA sequences of *SHISA3* and *RFC3* (Supplementary Table S1). To find single nucleotide polymorphisms (SNPs) in *SHISA3* and *RFC3*, 10 individual DNA samples from 1,382 sheep DNA samples were randomly chosen and blended as PCR templates for PCR fragment amplification and DNA sequencing. The PCR reaction that was done in order to sequence the DNA was carried out in a volume of reaction that was 25 ul, and it included 10 ul of PCR buffer, 0.35 uM of primers, 87.5 uM of dNTPs, 50 ng of genomic DNA, and 1.25 ul of UTaq DNA Polymerase (TransGen Biotech, Beijing, China). Using the following thermocycling conditions: 5 mins at 94°C, followed by 30 s at 94°C, followed by 30 s at 50–60°C, and finally 30 s at 72°C (35 cycles), followed by a final extension incubation for 5 mins at 72°C. The technique of competitive allele-specific FRET-based PCR analysis (KASPar) was then performed to genotype SNPs within *SHISA3* and *RFC3* (47). Information on primer pairs used for genotyping is listed in Supplementary Table S2. The 1,382 lambs were genotyped for *SHISA3* and *RFC3*, and finally 1,355 were successfully genotyped for *SHISA3*, while 1340 for *RFC3*.

### 2.4. Statistical analysis

In this study, all variables were subjected to the Shapiro-Wilk normality test (48), and those that failed the test were transformed using the logarithm (49). The general linear model was used to perform association analysis for various genotypes and FCR-related traits. The stats package of R (version 4.0.5) was used to model building which based on ordinary least square and execute significance tests. Genotype frequency and allele frequency, effective allele number (Ne), expected heterozygosity (He), expected homozygosity (Ho), and polymorphism information content (PIC) were calculated by referring to previous studies (50). The specific model and parameters are shown below:


$$\begin{array}{l} \;Y_{ijkl}=\mu +Genotype_{i}+Batch_{j}+Season_{k}+Sire_{l}+\;\varepsilon_{ijkl};\\ Y_{imjkln}=\mu +Genotype_{i}+Genotype_{m}+Batch_{j}+Season_{k}\\ \;+Sire_{l}+Combination_{n}+\;\varepsilon_{imjkln}, \end{array}$$

where Y_ijkl_ and Y_imjkln_ was the phenotypic observation value of FCR-related traits, μ is the mean, Genotype_i_ and Genotype_m_ is the effect of the ith and mth genotypes; Batch_j_ is the fixed effects (j = 1, 2,…, 6); Season_k_ is the fixed effects (k = 1, 2); Sire_l_ is the fixed effects; Combination_n_ refers to the effect of combination, ε_ijkl_ and ε_imjkln_ are the random error. All of our samples are collected around August (summer), and January (winter) of each year. To test the significance of the genotype means, the least significant difference (LSD) test was utilized. When *P* < 0.05 indicates statistical significance.

### 2.5. Total RNA preparation, and cDNA synthesis

Six sheep were selected at random from all of the samples (*n* = 1,382) to act as experimental subjects. Following that, these individuals' heart, liver, spleen, lung, kidney, muscle, tail fat, lymph, rumen, and duodenum were all collected. TRIzol reagent (Invitrogen, Waltham, MA, USA) was used to extract total RNA from the samples, which was then reverse transcribed into cDNA using a reverse transcriptase kit (TransGen Biotech, Beijing, China).

### 2.6. Analysis of *SHISA3* and *RFC3* expression levels

The mRNA levels of *SHISA3* and *RFC3* genes were detected in 10 tissues of the 6 sheep collected above. The Oligo 7.0 tool was used to design the particular primers utilized to evaluate gene expression (Supplementary Table S3). The qRT-PCR system that was used had a volume of 20 μl and included 10 μl of Takara Biotechnology's 2 X SYBR Green PCR Master Mixture, 0.8 μl of each primer, 2 μl of template cDNA, and 6.4 μl of RNase-free water (TransGen Biotech, Beijing, China). The conditions were as follows: 95°C for 3 mins, 95°C for 15 s, the optimal annealing temperature for 15 s, 72°C for 20 s, 40 cycles, and then 72°C for 5 mins. The qRT-PCR reactions were carried out using a Roche LightCycler 480 (Roche Applied Science, Basel, Switzerland): reaction protocols are based on earlier research (46). The data obtained were normalized using β-actin as an internal reference gene and analyzed using the 2^−ΔΔCt^ method (51). The Games-Howell test is a statistical model for measuring the degree of gene expression (52).

## 3. Results

### 3.1. Descriptive statistics on traits correlated with FCR

The results of the Shapiro-Wilk normality test showed that all traits conformed to the normal distribution, except for FCR80-140, which did not conform to the normal distribution. FCR80-140 was then log-transformed. Descriptive statistics are reported in Table 1. The genotyping sample size for Hu sheep was 1,382. The table shows the mean, standard deviation, maximum and minimum values for sheep BW80, BW100, BW120 and BW140. The average weight increase from BW80 to BW100, BW100 to BW120, and BW120 to BW140 for the bearded sheep population was 5.51 kg. The disparity between the maximum and minimum body weight readings increased with time, with a difference of 37.55 kg at BW140, with the greatest value being about three times the minimum.

**Table 1:** Descriptive statistics on traits correlated with FCR for *SHISA3* and *RFC3* genes.

**Item**	**Batch**	**BW80**	**BW100**	**BW120**	**BW140**	**FI80-140**	**ADFI80-140**	**ADG80-140**	**FCR80-140**
No.	1	97	97	97	97	97	97	97	97
	2	205	205	205	205	205	205	205	205
	3	165	165	165	165	165	165	165	165
	4	314	314	314	314	314	314	314	314
	5	180	180	180	180	180	180	180	180
	6	421	421	421	421	421	421	421	421
Mean	1	19.59	24.56	30.34	35.74	82.72	1.38	0.27	5.16
	2	22.28	27.59	33.10	38.53	87.02	1.45	0.27	5.46
	3	18.80	23.78	29.19	34.51	76.17	1.27	0.26	4.91
	4	21.49	27.30	32.93	37.86	90.92	1.52	0.27	5.66
	5	19.39	24.86	30.76	37.11	90.46	1.51	0.30	5.15
	6	17.46	22.27	28.20	34.19	83.67	1.39	0.28	5.04
SD	1	3.42	4.07	4.52	4.89	12.75	0.21	0.04	0.59
	2	3.57	4.03	4.51	4.99	12.57	0.21	0.05	0.84
	3	2.99	3.47	3.65	3.96	10.01	0.17	0.04	0.66
	4	3.94	4.65	5.34	5.86	15.66	0.26	0.06	0.85
	5	3.53	4.12	4.58	5.23	13.94	0.23	0.05	0.65
	6	3.71	4.70	5.36	5.88	16.68	0.28	0.05	0.65
Max	1	27.50	33.70	40.80	48.30	111.60	1.86	0.36	7.19
	2	32.50	38.80	45.60	54.10	121.70	2.03	0.42	10.24
	3	30.10	35.50	40.10	45.50	102.70	1.71	0.46	7.76
	4	33.90	42.40	49.40	57.10	134.75	2.25	0.43	10.66
	5	30.35	37.15	42.15	50.25	125.80	2.10	0.42	8.12
	6	28.40	35.60	44.00	52.50	133.30	2.22	0.45	11.22
Min	1	11.30	13.40	18.20	21.00	45.50	0.76	0.15	3.27
	2	14.40	16.40	20.60	24.00	52.00	0.87	0.12	3.26
	3	12.50	15.80	20.00	25.60	54.00	0.90	0.15	3.19
	4	11.80	15.20	20.60	23.25	50.95	0.85	0.10	4.12
	5	11.85	14.45	18.00	23.70	56.90	0.95	0.17	3.74
	6	9.50	9.78	13.80	19.55	38.00	0.63	0.08	3.74

BW80, 80 days body weight; BW100, 100 days body weight; BW120, 120 days body weight; BW140, 140 days body weight; FI80-140, feeding intake at 80–140 days; ADG80-140, average daily weight gain from 80 to 140 days; FCR80-140, feed conversion ratio at 80–140 days.

The unit of BW is KG.

The unit of FI is KG.

The unit of ADG is KG/d.

### 3.2. SNP scanning of *SHISA3* and *RFC3* in Hu sheep

**Figure 1:**
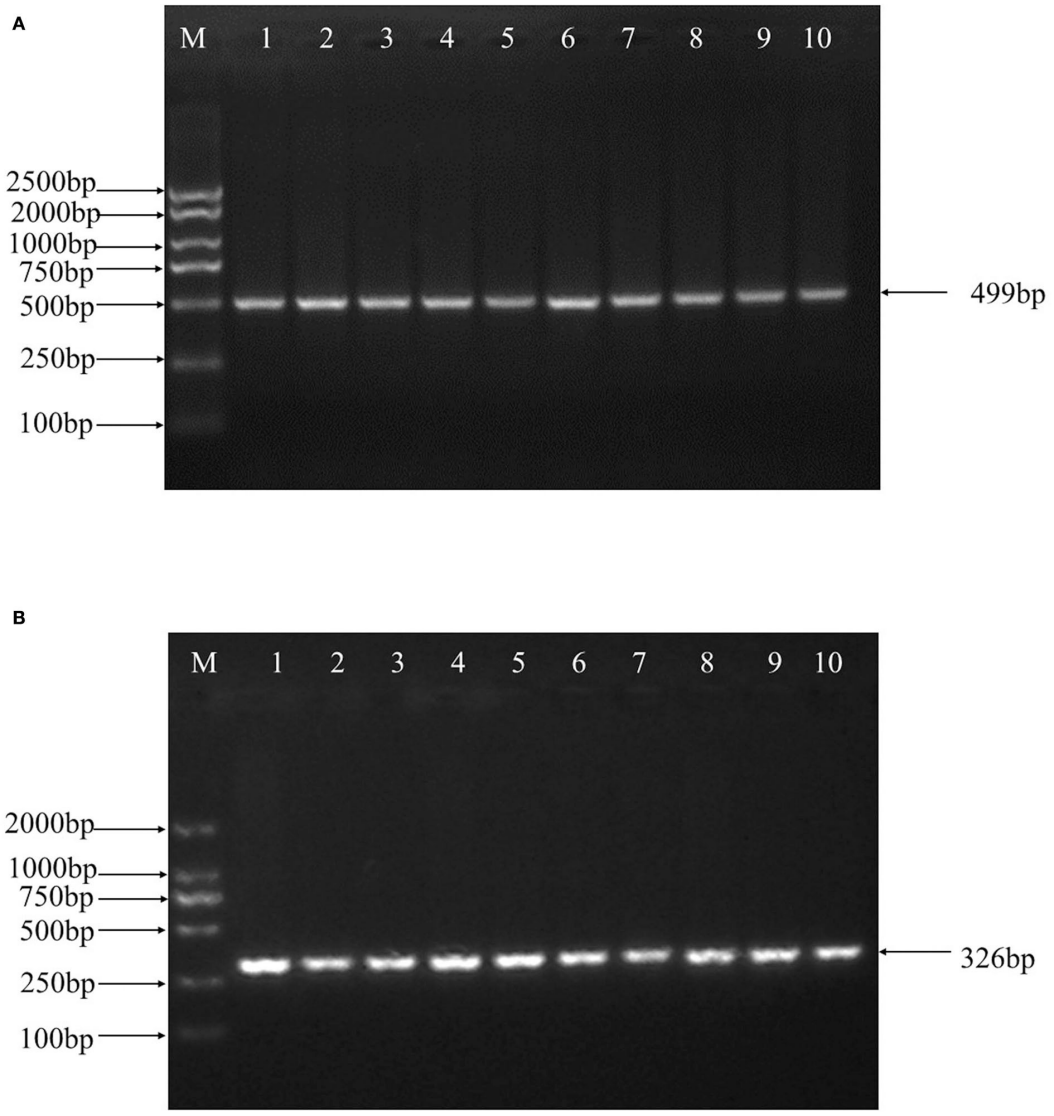
PCR amplification of ovine *SHISA3* **(A)** and *RFC3* **(B)** genes target fragment. M, DNA marker; 1–10, PCR products.

**Figure 2:**
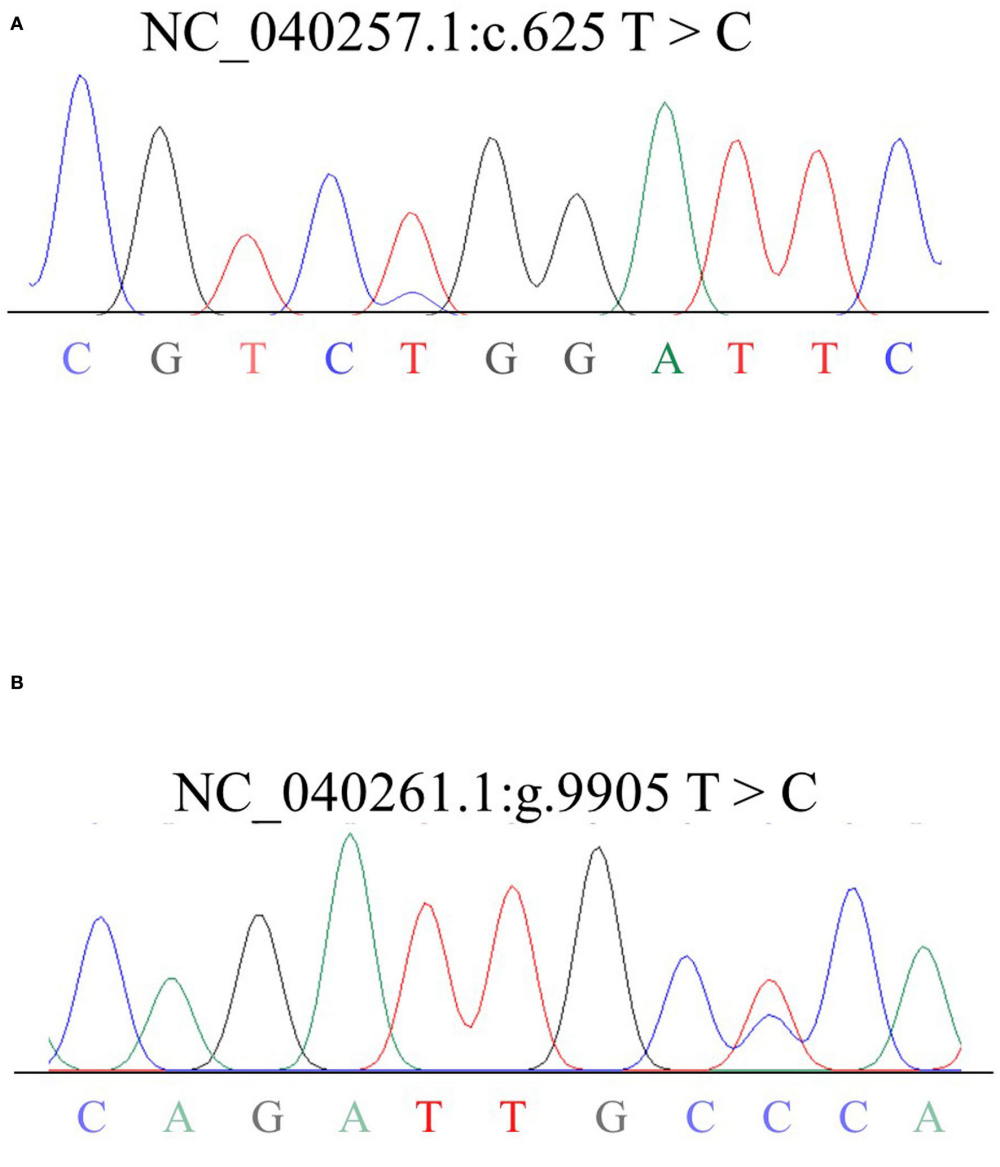
Sequencing results of mixed DNA from Hu sheep *SHISA3* **(A)** and *RFC3* **(B)**. Overlapping peaks reflect mutation sites.

**Figure 3:**
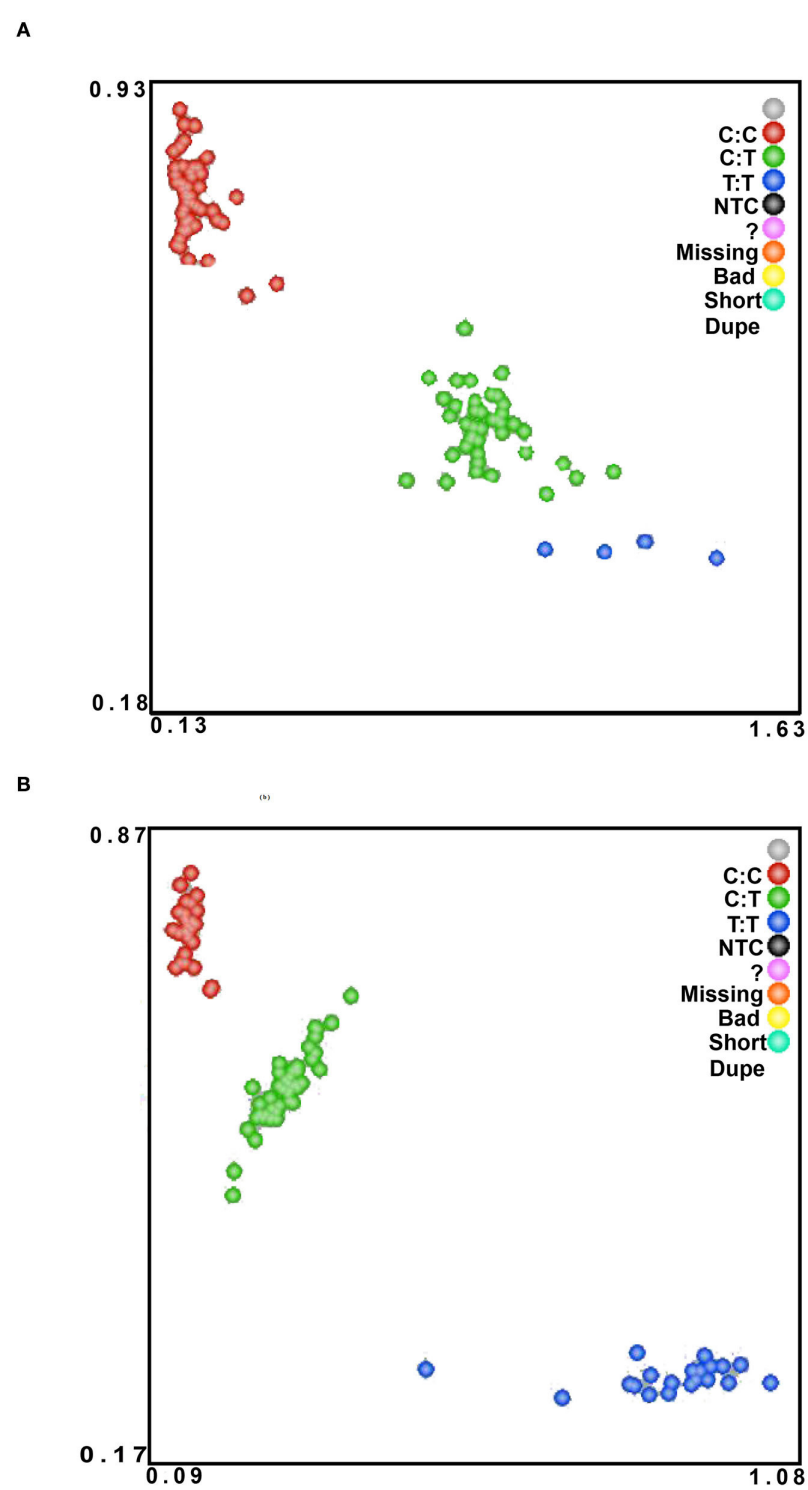
The KASPar method was used to genotype Hu sheep NC_040257.1:c.625 T > C **(A)** and NC_040261.1:g.9905 T > C **(B)** SNPs in the experimental population. The color of the dots indicates the result of genotyping; green, red, and blue in the figure indicate three different genotypes.

499 bp and 326 bp PCR fragment sequence were amplified from the DNA pool of the experimental population using the primer pairs shown in Supplementary Table S1, Figure 1 and each detected a mutant site (Figure 2). Both mutations were genotyped using the KASPar method and all three genotypes, CC, CT and TT, were detected (Figure 3). The genotype frequencies, allele frequencies, and genetic diversity of the two genes are displayed in Table 2. The frequencies of the three genotypes CC, CT and TT for NC_040257.1:c.625 T > C and NC_040261.1:g.9905 T > C were 0.46, 0.46, 0.08 and 0.30, 0.49, 0.21, respectively. The gene frequency of C in NC_040257.1:c.625 T > C was 0.69, whereas it was 0.54 in NC_040261.1:g.9905 T > C. The Ne, Ho, He, and PIC values for the *SHISA3* gene were 1.75, 0.57, 0.43, and 1.75, respectively, and for the *RFC3* gene were 2, 0.5, 0.5, and 0.38 (Table 2), indicating that the two genes showed intermediate polymorphism.

**Table 2:** The genotype frequency, allele frequency and genetic diversity of *SHISA3* and *RFC3* sites.

**Loci**	**Genotype**	**Genotype frequency**	**Allele**	**Allele frequency**	**Ne**	**Ho**	**He**	**PIC**
**NC_040257.1:c.625 T** **>** **C**	CC (627)	0.46	C	0.69	1.75	0.57	0.43	0.34
	CT (620)	0.46						
	TT (108)	0.08	T	0.31				
**NC_040261.1:g.9905 T** **>** **C**	CC (395)	0.30	C	0.54	2	0.5	0.5	0.38
	CT (660)	0.49						
	TT (285)	0.21	T	0.46				

Ne, effective allele number; Ho, expected homozygosity; He, expected heterozygosity; PIC, polymorphism information content.

### 3.3. Association analysis of Hu sheep *SHISA3* and *RFC3* with traits related to feed conversion ratio

To study the effects of the SNPs of Hu sheep NC_040257.1:c.625 T > C and NC_040261.1:g.9905 T > C on FCR, association analysis was performed using the linear model in R 4.0.5 version. The results indicated that the SNP at NC_040257.1:c.625 T > C was significantly associated with BW80, 100, 120 (*P* < 0.05) (Table 3). BW80 and BW100 were significantly higher in animals carrying the CC genotype than in those carrying the CT or TT genotypes (*P* < 0.05). However, at BW120, the CC genotype outperformed the TT genotype, while the CT genotype did not differ from the other two genotypes (*P* > 0.05). The FCR80-140 association results revealed that CT and TT genotypes performs better CC genotypes. The findings of the association analysis for SNP NC_040261.1:g.9905 T > C revealed no significant effects with body weight and feed intake (*P* > 0.05). The results of the ADG80-140 and FCR80-140 association analysis revealed that the CC genotype with NC_040261.1:g.9905 T > C was markedly better than the TT genotype (*P* < 0.05).

**Table 3:** The association between *SHISA3* and *RFC3* polymorphism and traits related to feed conversion ratio.

	**NC_040257.1:c.625 T** > **C**			***P***	**NC_040261.1:g.9905 T** > **C**			***P***
**Item**	**CC**	**CT**	**TT**		**CC**	**CT**	**TT**	
**No**.	627	620	108		395	660	285	
BW80	19.941 ± 1.762^a^	19.433 ± 1.731^ab^	19.256 ± 1.662^b^	0.010	19.629 ± 1.798	19.646 ± 1.759	19.656 ± 1.747	0.883
BW100	25.172 ± 2.045^a^	24.656 ± 2.000^b^	24.507 ± 1.932^b^	0.031	24.851 ± 2.062	24.872 ± 2.024	24.878 ± 2.007	0.924
BW120	30.913 ± 1.909^a^	30.371 ± 1.864^b^	30.165 ± 1.808^b^	0.036	30.627 ± 1.923	30.584 ± 1.892	30.525 ± 1.874	0.782
BW140	36.469 ± 1.522	35.998 ± 1.483	35.788 ± 1.446	0.083	36.386 ± 1.536	36.169 ± 1.514	35.936 ± 1.500	0.259
FI80-140	86.538 ± 3.268^a^	85.251 ± 3.246^ab^	84.062 ± 3.669^b^	0.043	86.072 ± 3.283	85.718 ± 3.359	85.225 ± 3.499	0.446
ADFI80-140	1.443 ± 0.054^a^	1.421 ± 0.054^ab^	1.402 ± 0.061^b^	0.049	1.435 ± 0.055	1.429 ± 0.056	1.421 ± 0.058	0.480
ADG80-140	0.276 ± 0.005	0.277 ± 0.005	0.276 ± 0.005	0.978	0.280 ± 0.006^a^	0.276 ± 0.005^ab^	0.272 ± 0.006^b^	0.029
FCR80-140	5.307 ± 0.256^a^	5.211 ± 0.248^b^	5.147 ± 0.255^b^	0.009	5.190 ± 0.257^b^	5.255 ± 0.258^b^	5.314 ± 0.257^a^	0.022

The unit of BW is KG. The unit of FI is KG. The unit of ADG is KG/d. Significant differences in the same row are denoted by distinct lowercase letters (P < 0.05).

### 3.4. Association analysis of combined genotypes of *SHISA3* and *RFC3* genes with traits related to feed conversion ratio

The combined impacts of various genotypes of the NC_040257.1:c.625 T > C and NC_040261.1:g.9905 T > C polymorphisms with attributes associated to FCR were evaluated using linear regression analysis (Table 4). As both NC_040257.1:c.625 T > C and NC_040261.1:g.9905 T > C have three genotypes, a three-by-three combinatorial pattern was generated. BW80, 100, 120, 140, FI80-140, and ADG80-140 did not present significance in association analysis of various combinations (*P* > 0.05). However, in FCR80-140, TT^*SHISA*3^-CC^*RFC*3^, TT^*SHISA*3^-TT^*RFC*3^, TT^*SHISA*3^-CT^*RFC*3^, CT^*SHISA*3^-CC^*RFC*3^ and CT^*SHISA*3^-CT^*RFC*3^ showed better results than CC^*SHISA*3^-TT^*RFC*3^ (*P* < 0.05) (Table 4).

**Table 4:** Association analysis of combined genotypes at the *SHISA3* and *RFC3* traits related to feed conversion ratio.

**Item**	**Genotype**								
	**CC** *^*SHISA*3^* **-TT** *^*RFC*3^*	**CT** *^*SHISA*3^* **-TT** *^*RFC*3^*	**CC** *^*SHISA*3^* **-CT** *^*RFC*3^*	**CC** *^*SHISA*3^* **-CC** *^*RFC*3^*	**CT** *^*SHISA*3^* **-CT** *^*RFC*3^*	**CT** *^*SHISA*3^* **-CC** *^*RFC*3^*	**TT** *^*SHISA*3^* **-CT** *^*RFC*3^*	**TT** *^*SHISA*3^* **-TT** *^*RFC*3^*	**TT** *^*SHISA*3^* **-CC** *^*RFC*3^*
No.	122	141	312	184	288	180	55	20	30
W80	20.041 ± 1.818	19.891 ± 1.737	19.707 ± 1.759	19.369 ± 1.715	19.422 ± 1.738	19.337 ± 1.747	19.519 ± 1.447	19.225 ± 1.770	19.121 ± 1.693
BW100	25.29 ± 2.100	25.124 ± 2.018	24.919 ± 2.026	24.567 ± 1.972	24.642 ± 2.005	24.555 ± 2.013	24.787 ± 1.750	24.433 ± 2.030	24.351 ± 1.960
BW120	31.06 ± 1.964	30.871 ± 1.894	30.647 ± 1.893	30.299 ± 1.842	30.34 ± 1.875	30.226 ± 1.881	30.343 ± 1.678	30.08 ± 1.893	29.984 ± 1.837
BW140	36.641 ± 1.569	36.458 ± 1.520	36.247 ± 1.512	35.954 ± 1.469	35.956 ± 1.499	35.835 ± 1.502	35.829 ± 1.392	35.686 ± 1.507	35.592 ± 1.474
FI80-140	87.096 ± 3.213	86.436 ± 3.425	85.756 ± 3.498	85.459 ± 3.354	85.161 ± 3.358	84.646 ± 3.267	83.409 ± 4.878	84.04 ± 3.371	84.005 ± 3.624
ADFI80-140	1.452 ± 0.053	1.441 ± 0.057	1.43 ± 0.058	1.425 ± 0.056	1.42 ± 0.056	1.411 ± 0.054	1.391 ± 0.081	1.401 ± 0.056	1.401 ± 0.060
ADG80-140	0.277 ± 0.006	0.277 ± 0.005	0.276 ± 0.006	0.277 ± 0.006	0.276 ± 0.006	0.276 ± 0.006	0.272 ± 0.006	0.275 ± 0.006	0.275 ± 0.006
FCR80-140	5.313 ± 0.249^a^	5.282 ± 0.249^ab^	5.247 ± 0.244^ab^	5.213 ± 0.236^ab^	5.213 ± 0.241^ab^	5.193 ± 0.240^b^	5.182 ± 0.271^b^	5.167 ± 0.240^b^	5.163 ± 0.244^b^

The unit of BW is KG. The unit of FI is KG. The unit of ADG is KG/d. Different superscript letters represent a statistically significant difference between categories compared, and same superscript letters indicate no statistical difference between categories compared.

### 3.5. Analysis of *SHISA3* and *RFC3* expression levels in the different tissues

**Figure 4:**
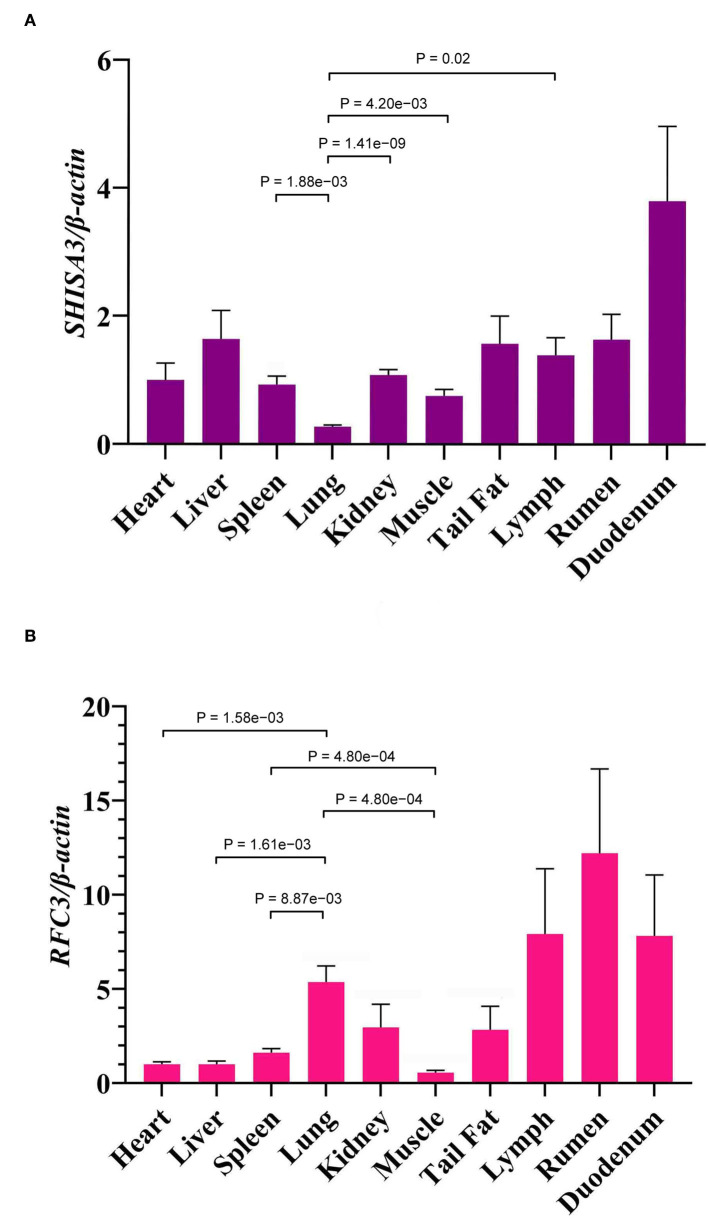
The expression of *SHISA3* mRNA in different tissues **(A)**. The expression of *RFC3* mRNA in different tissues **(B)**.

The expression levels of *SHISA3* (Figure 4A) and *RFC3* (Figure 4B) genes in heart, liver, spleen, lung, kidney, rumen, duodenum, muscle, lymph and tail fat were analyzed by qRT-PCR, and the results showed significant differences in expression levels in different tissues (*P* < 0.05) (Figure 4). Figure 4A shows the expression levels of the *SHISA3* gene, which were significantly lower in the lung than in spleen, kidney, muscle and lymph (*P* < 0.05). However, the expression pattern of *RFC3* differed from that of the *SHISA3* gene (Figure 4B). Notably, the lung was extremely significant more expressed than the heart, liver, spleen and muscle (*P* < 0.01), while spleen was also extremely significantly higher than muscle (*P* < 0.01). It is also widely distributed in the lymph, rumen and duodenum.

## 4. Discussion

The FCR has a considerable impact on agricultural efficiency, economic viability, and environmental sustainability (53). FCR is impacted by a variety of variables, making it difficult for breeding programs to test it directly on candidate populations (54). Broiler dietary protein and energy sources have a major influence on FCR (55), and beef cattle live weight affects FCR through influencing maintenance and production needs, according to studies (56). In this study, we compared different stages of ADG to observe changes in weight and FCR-related characteristics over time. The findings revealed that body weight did not correlate with ADG but did associate with FCR. FCR features are economically significant qualities that have been the focus of the growing livestock sector, and advances in FCR directly lead to feed consumption reductions (57). These results show that FCR is a measurable trait that may be used to direct breeding and selection.

SNPs are single alterations in a single base in the genome that most typically describe inter-individual genetic variation (58). Because SNPs are associated with numerous economically relevant features in livestock, they have grown in importance as a molecular marker (59). It may be separated into synonymous mutations, missense mutations, and silent mutations based on the various kinds of mutation (60). Missense mutations were more likely to occur than silent mutations (61). Numerous studies have shown that missense mutations may play a crucial role in the development of many illnesses (62). In this work, we identified a missense mutation in *SHSA3*. The results show that it is associated with body weight and feed efficiency in sheep. Silent mutations are often ignored because they do not result in changes to the proteins (63). However, synonymous mutations have their own benefits in terms of modifying mRNA stability, splicing regulatory regions, miRNA binding sites, or translation efficiency, all of which result in changes in protein level or shape (64). Recent research has shown that synonymous or silent mutations contribute to the breeding of economic features in sheep (65–67).

In this study, two SNPs were discovered in NC_040257.1:c.625 T > C and NC_040261.1:g.9905 T > C. The potential of an association between these two mutant loci and BW, FI, ADG, and FCR was studied. The results showed that NC_040257.1:c.625 T > C was significantly associated with BW80, BW100, BW120 and FCR (*P* < 0.05). NC_040261.1:g.9905 T > C was significantly associated with ADG and FCR (*P* < 0.05). *SHISA3* has been demonstrated in studies to contribute to the suppression of carcinogenesis, invasion, and metastasis by increasing β-catenin degradation (29). The *RFC3* gene is implicated in the enrichment of the Wnt/-catenin signaling pathway (34). The Wnt pathway has been found to play a key part in its formation as well as its anti-inflammatory properties (68, 69). Animal FE is related to immunoinflammatory effects (21), hence we predicted that NC_040257.1:c.625 T > C and NC_040261.1:g.9905 T > C is associated to animal feed efficiency.

Association analysis revealed a strong correlation between the combined genotypes of these two loci (NC_040257.1:c.625 T > C and NC_040261.1:g.9905 T > C) and the FCR of Hu sheep (*P* < 0.05). In addition, FCR increases with increasing T allele frequency and peaks when both genes are for T. The mRNA level of *SHISA3* was substantially greater in the duodenum than in other organs, according to the findings of qRT-PCR analysis. The intestine is a vital digestive organ and the greatest immunological organ in animals, and the duodenum, as a component of the intestine, likewise performs digestive and immune activities. In addition, duodenal bypass is essential for improving glycemic control (70). As a result, our hypothesis that the immune response is linked to animal feed efficiency has been confirmed. Additionally, lymph, rumen, and duodenum had significantly higher *RFC3* expression levels than other tissues. When this study's expression profile was compared to the bovine gene expression profile, the findings were very similar (71). Animal rumen efficiency is related to feed efficiency, according to studies, and rumen passage rate is affected by both feed intake and rumen size (72–74). Furthermore, both the lymph and the duodenum are immunological tissues that execute immune tasks in the animal (75, 76). As a consequence, we speculated that mutations in the *SHISA3* and *RFC3* genes may influence animal FE by affecting the immunological response. The *SHISA3* and *RFC3* genes in sheep may be chosen as candidate genes for improved FCR. However, further research is required to confirm the association between *SHISA3* and *RFC3* and FE features.

## 5. Conclusion

In short, NC_040257.1:c.625 T > C and NC_040261.1:g.9905 T > C were shown to be substantially associated with feed efficiency features in this research (BW, ADG, FCR). In the sheep population, these two genes had a similar influence on FCR. *SHISA3* expression was significantly higher in the duodenum, while *RFC3* expression was significantly higher in the rumen, lymph, and duodenum than in the other tissues. As a conclusion, using the *SHISA3* and *RFC3* genes as genetic markers might help to enhance FCR while also increasing economic efficiency.

## Data availability statement

The original contributions presented in the study are included in the article/Supplementary material, further inquiries can be directed to the corresponding authors.

## Ethics statement

The animal study was reviewed and approved by Gansu Agricultural University's Animal Health and Ethics Committee.

## Author contributions

XZh, CL, and WZ designed the study. XL, YZhao, JW, JC, DX, WL, BZ, and LZ involved in animal husbandry and liver sample collection. ZM, XY, XZe, JL, RZ, PC, and YH involved in DNA extraction. CL wrote the paper. YZhan, DZ, KH, WWa, LS, XW, and WWu reviewed and edited the manuscript. All authors contributed to the article and approved the submitted version.
