# Supplementary material for: Polymorphisms in SHISA3 and RFC3 genes and their association with feed conversion ratio in Hu sheep
Source: Front Vet Sci. 2023 Jan 5;9:1010045. doi: 10.3389/fvets.2022.1010045 (PMC9850526; doi:10.3389/fvets.2022.1010045)
Supplement: Supplementary file 1 [file Data_Sheet_1.PDF]

Table S1. Primer sequences and PCR parameters.

Gene name	accession number	Sense primer (5'–3')	Anti-sense primer (5'–3')	Size	Tm (℃)
SHISA3	NC_040257.1	TCACTCCGCAGCTATCAGAC	ATTTTCCTCATACAGCCGTTC	499bp	55℃
RFC3	NC_040261.1	CTGATAGCTAGGTTAGACCCC	TAACTGACCCCTTTTACTCAC	326bp	55℃

Table S2. Primers for the KASPar assay.

Gene name	Primer name	accession number	Primer sequence (5'–3')
SHISA3	Primer_AlleleX	NC_040257.1	GAAGGTGACCAAGTTCATGCTTGGGGTGACACCAGGAATCCA
	Primer_AlleleY		GAAGGTCGGAGTCAACGGATTGGGGTGACACCAGGAATCCG
	Primer_Common		CAGGCCACCCAATCCACTTGAC
RFC3	Primer_AlleleX	NC_040261.1	GAAGGTGACCAAGTTCATGCTTCTCAGCATATAGTTTAACAGATTGCT
	Primer_AlleleY		GAAGGTCGGAGTCAACGGATTCTCAGCATATAGTTTAACAGATTGCC
	Primer_Common		AACACATGGCAAATCTACAAATAAAATAATCAAC

Table S3. Primer pairs used to qRT-PCR for the ovine *SHISA3* and *RFC3* genes.

Gene name	accession number	Primer sequence (5'–3')	Sense primer (5'–3')	Size	Tm (℃)
SHISA3	XM_015096480.3	CGCAGCCTGTCTACGTTCCCT	TCTCTGTCTGATAGCTGCGGAGT	159 bp	57℃
RFC3	XM_004012131.5	AGCATTGAAGATATTTGCCAT	GACAATAGCATTGGCAGTCTC	228 bp	55℃
β-actin	NM_001009784.3	TCCGTGACATCAAGGAGAAGC	CCGTGTTGGCGTAGAGGT	267 bp	60℃
